# Attitudes to ageing, biomarkers of ageing and mortality: the Lothian Birth Cohort 1936

**DOI:** 10.1136/jech-2019-213462

**Published:** 2020-01-28

**Authors:** Kyle J J McLachlan, James H Cole, Sarah E Harris, Riccardo E Marioni, Ian J Deary, Catharine R Gale

**Affiliations:** 1 Medical School, The University of Edinburgh, Edinburgh, UK; 2 Neuroimaging, Institute of Psychiatry, Psychology and Neuroscience, London, UK; 3 Centre for Medical Imaging Computing, Computer Science, University College London, London, UK; 4 Dementia Research Centre, Institute of Neurology, University College London, London, UK; 5 Psychology, The University of Edinburgh, Edinburgh, UK; 6 MRC Institute of Genetics and Molecular Medicine, The University of Edinburgh, Edinburgh, UK; 7 MRC Lifecourse Epidemiology Unit, University of Southampton, Southampton, Hampshire, UK

**Keywords:** mortality, epidemiology of ageing, psychology

## Abstract

**Objective:**

To investigate whether people with more positive attitudes to ageing are biologically younger as defined by leucocyte telomere length, accelerated DNA methylation GrimAge (AgeAccelGrim) and brain-predicted age difference, and whether these biomarkers explain relationships between attitudes to ageing and mortality.

**Methods:**

We used linear regression to examine cross-sectionally attitudes to ageing (measured using the Attitudes to Ageing Questionnaire) and the three biomarkers in 758 adults, mean age 72.5 years, from the Lothian Birth Cohort 1936. We used Cox proportional hazards models to examine longitudinally attitudes to ageing and mortality and the role of the biomarkers.

**Results:**

More positive attitude to physical change was associated with younger biological age, as measured by AgeAccelGrim and brain-predicted age difference in age-adjusted and sex-adjusted models: for an SD higher score, AgeAccelGrim was lower by -0.73 (95% CI -1.03 to -0.42) of a year, and brain-predicted age difference was lower by -0.87 (1.51 to 0.23) of a year. Both associations were attenuated by adjustment for covariates and not significant after simultaneous adjustment for all covariates and correction for multiple testing. More positive attitudes to physical change were associated with lower mortality: for an SD higher score the age-adjusted and sex-adjusted HR (95% CI) was 0.66 (0.56 to 0.78). Adjustment for AgeAccelGrim or brain-predicted age difference attenuated this association slightly. It remained significant after adjustment for all covariates.

**Conclusion:**

We found partial evidence that attitudes to ageing are linked with ageing biomarkers but they accounted for only a little of the association between attitudes and mortality.

## Introduction

The rapid rate of population ageing has motivated research into influences on health and longevity of older people. Attitudes to ageing, which encompass personal experiences of growing old and general beliefs about ageing,^1^ is one such factor. Levy hypothesises that older people internalise cultural age stereotypes—which often associate ageing with physical decline, disability and loneliness—such that they become a self-fulfilling prophecy.^2^ Having more negative perceptions of ageing has been associated with a range of adverse health outcomes.^3–7^ A meta-analysis found that having a younger subjective age is associated with better health outcomes and a longer life.^8^ Negative perceptions of ageing have been linked with higher mortality.^9–11^

The underlying mechanisms are poorly understood, but cardiovascular stress,^12^ cortisol levels,^13^ inflammatory biomarkers^14^ and health behaviours may play a role. In 335 older adults, those with negative views of ageing had a shorter telomere length 4 years later than those with positive attitudes.^15^ Age-related structural brain changes are also more advanced in people with an older subjective age.^16^ These findings suggest that attitudes to ageing may be associated with cellular and brain ageing.

Telomere length, DNA methylation age and brain-predicted age difference (brain-PAD) are biomarkers of ageing.^17^ Leucocyte telomere length decreases each time a cell replicates and has thus been considered a marker of cellular ageing. DNA methylation occurs throughout the genome at cytosine-phosphate-guanine (CpG) sites to regulate gene expression. Methylation patterns give an accurate prediction of chronological age which can be used to calculate a novel measure of biological ageing known as ‘accelerated DNA methylation GrimAge’ (AgeAccelGrim),^18^ which was trained to predict survival. Brain-PAD is derived from brain MRI scan data. Structural brain changes can be identified, which suggest whether an individual’s ‘brain age’ is younger or older than their actual age.^17^ All three biomarkers are independent predictors of mortality,^19 20^ and given that attitudes to ageing are associated with telomere length^15^ and subjective age is associated with brain-PAD,^16^ they may help explain the relationship between attitudes to ageing and mortality in older people.

We investigated whether people with more positive attitudes to ageing are biologically younger as defined by leucocyte telomere length, AgeAccelGrim and brain-PAD, and examined the extent to which any relationship between attitudes to ageing and mortality might be explained by the ageing biomarkers.

## Methods

### Participants

The Lothian Birth Cohort 1936 (LBC1936) consists of surviving participants of the 1947 Scottish Mental Survey recruited to investigate healthy ageing.^21 22^ At wave 1, 1091 people (mean age 70 years) were recruited. This study uses data from wave 2 (mean age 72.5, range 70.9–73.4) and mortality data from date of the wave 2 assessment to April 2018.^22^ Participants gave written informed consent.

### Measures

#### Attitudes to ageing

Participants completed the ‘Attitudes to Ageing Questionnaire’ (AAQ)^1^ by post at about the same time as wave 2 clinic testing. It consists of 24 items scored on a 5-point Likert scale which capture general attitudes towards the ageing process and personal experience of ageing.^1^ Scores are calculated under three domains: physical change, psychosocial loss and psychological growth. Examples of items in each domain include: “*my health is better than I expected for my age”, ‘old age is a time of loneliness’* and *‘wisdom comes with age’*. More positive attitudes to ageing are indicated by higher scores for physical change and psychological growth, and lower scores for psychosocial loss.

#### Biomarkers of ageing

##### Leucocyte telomere length

Leucocyte telomere length was measured from DNA extracted from whole blood samples at wave 2^23^ followed by quantitative PCR, using an Applied Biosystems (Pleasonton, California, USA) 7900HT Fast Real-Time PCR machine. Four internal control DNA samples were run within each plate to correct for plate-to-plate variation.

##### Epigenetic age acceleration

Illumina HumanMethylation 450 BeadChips were used to measure DNA methylation from blood samples at wave 2. DNA methylation occurs throughout the genome at CpG sites to regulate gene expression. Methylation patterns give an accurate prediction of chronological age.^24 25^ There are several DNA methylation-based biomarkers which are used to measure epigenetic age or epigenetic age acceleration, known as the ‘epigenetic clock’, one of which is ‘DNA methylation GrimAge’.^18^ This is a novel epigenetic clock which combines age, sex, DNA methylation-based surrogates for smoking and the levels of seven serum proteins.^26^ As is the case with other epigenetic clocks, the difference between DNA methylation GrimAge and chronological age—accelerated DNA methylation GrimAge (AgeAccelGrim)—provides a measure of biological ageing. This variable was derived by taking residuals from a linear regression model of DNA methylation GrimAge on chronological age. Details of how these data were collected and measured have been reported previously.^26–28^

##### Brain-predicted age difference

T1-weighted structural MRI scans at wave 2 were used to identify voxel-wise patterns of brain volume which indicate the degree of brain ageing^29^ and thus inform the calculation of ‘brain-predicted age’,^17^ by reference to a regression model defined in an independent sample of healthy individuals (n=2001, aged 18–90 years).^30^ Brain-PAD was calculated by subtracting actual age from ‘brain-predicted age’.^17^

Information on the variability of the biomarkers of ageing is provided in the online supplementary file 1.

10.1136/jech-2019-213462.supp1 Supplementary data

## Mortality

National Records of Scotland provided mortality data for the LBC1936 participants via data linkage with the National Health Service Central Register.

## Covariates

At wave 2, symptoms of depression and anxiety were measured using the Hospital Anxiety and Depression Scale.^31^ Participants were asked if they had been diagnosed with hypertension, cardiovascular disease, stroke, diabetes, cancer and arthritis and responses were combined to give a total number of chronic illnesses. Other covariates were number of years spent in full-time education, social class, smoking status, number of days alcohol is consumed per week and time taken to walk 6 metres.

## Statistical analysis

We used rank-order correlations to examine bivariate associations between leucocyte telomere length, AgeAccelGrim and brain-PAD and other characteristics. Point biserial correlations were used for characteristics which were binary variables.

Linear regression was used to examine cross-sectional relationships between each domain of attitudes to ageing and each ageing biomarker, adjusting for age and sex and then further adjusting for other covariates. AAQ scores were standardised to mean 0 and SD 1 in order to facilitate comparisons. Leucocyte telomere length was log-transformed because it had a skewed distribution. All analyses of leucocyte telomere length and AgeAccelGrim were adjusted for measured white blood cell counts.

Cox proportional hazard regression was used to examine relationships between each domain of attitudes to ageing and risk of death, adjusting for age and sex, further adjusting for the other covariates then adding each biomarker of ageing in sequential models. Analyses of leucocyte telomere length and AgeAccelGrim were also adjusted for white blood cell counts. Survival time in days was calculated from date of the wave 2 survey to date of death or April 2018, whichever occurred first. Follow-up time ranged from 5.4 to 7.9 years (mean=6.8). We tested that the proportional hazards assumptions were met using Schoenfeld residuals.

As we carried out multiple tests of statistical significance, we corrected the p values in the multivariable models by applying the False Discovery Rate^32^ across the associations between the three ageing biomarkers and the three attitude to ageing domain scores and then across the associations between the three attitude to ageing domain scores and mortality.

## Results

**Figure 1:**
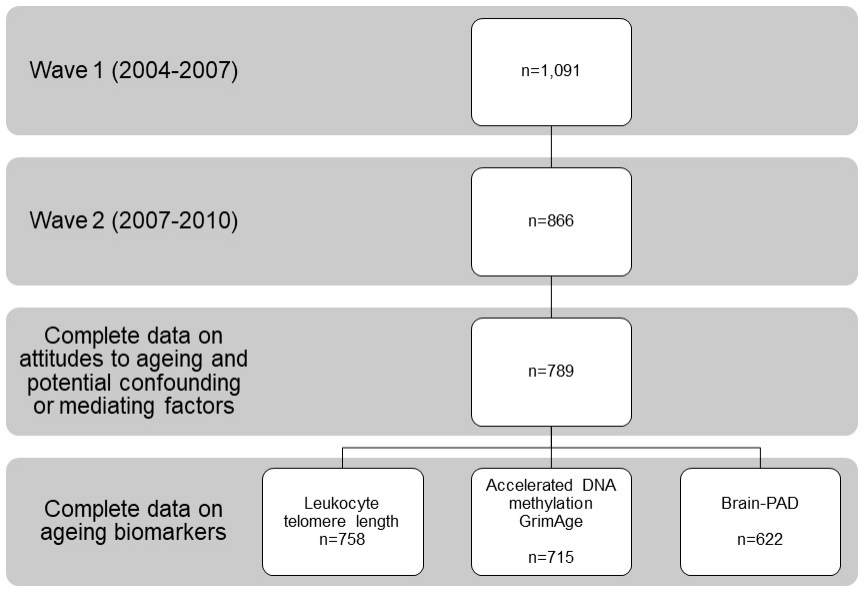
Sample sizes at waves 1 and 2. Brain-PAD, brain-predicted age difference.

Of the 866 participants in wave 2, 789 (91%) had complete data on attitudes to ageing and covariates. Of these 789 participants, 758 (96%) had data on leucocyte telomere length, 715 (91%) had data on AgeAccelGrim and 622 (79%) had data on brain-PAD (figure 1). Compared with these 622 participants, the 469 cohort members who were excluded from analyses because they did not take part in wave 2 or had missing data at wave 2, had spent slightly less time in full-time education, were less likely to have a non-manual social class and had higher scores for depression and more chronic disease at wave 1.

Table 1 describes the characteristics of the largest sample (n=758) and shows the rank-order correlations between these characteristics and the three biomarkers of ageing and death during follow-up. In these bivariate analyses, AgeAccelGrim was positively associated with being male, less education, lower social class, more chronic disease, more symptoms of depression, having a history of smoking, drinking alcohol more frequently, slower walking speed and having a more negative attitude to physical change and to psychosocial loss. AgeAccelGrim was negatively associated with symptoms of anxiety. Higher brain-PAD (more advanced brain ageing relative to chronological age) was associated with being male,^20^ more chronic disease, more symptoms of depression, smoking, drinking alcohol more frequently, having a more negative attitude to physical change. Higher brain-PAD was associated with fewer symptoms of anxiety. Shorter leucocyte telomere length was associated with being female and with older age, but there were no associations with attitudes to ageing. Of the three biomarkers of ageing, AgeAccelGrim and brain-PAD were positively correlated (rho=0.198), but neither were associated with leucocyte telomere length. Death during follow-up was associated with being male, lower social class, more chronic disease, greater depression, history of smoking, slower walking speed, being biological older as measured by brain-PAD^20^ and AgeAccelGrim,^26^ and with having more negative attitudes to ageing in all three domains.

**Table 1:** Characteristics of participants and their rank-order correlations with ageing biomarkers and mortality (n=758)

Characteristics	Mean (SD) or number (%)	Correlation with leucocyte telomere length	Correlation with accelerated DNA methylation GrimAge	Correlation with brain-PAD	Correlation with death during follow-up
		n=758	n=715	n=622	n=758
Age in years, mean (SD)	72.5 (0.71)	0.075 (p=0.039)	0.028 (p=0.463)	0.016 (p=0.695)	−0.046 (p=0.205)
Female, number (%)	364 (48.0)	−0.128 (p=0.004)	−0.459 (p<0.001)	−0.300 (p<0.001)	−0.111 (p=0.002)
Number of years in education, mean (SD)	10.8 (1.14)	−0.037 (p=0.312)	−0.131 (p=0.001)	0.012 (p=0.770)	−0.041 (p=0.268)
Non-manual social class,* number (%)	595 (78.5)	0.027 (p=0.464)	0.185 (p<0.001)	0.035 (p=0.385)	0.139 (p=0.0001)
Number of chronic diseases, mean (SD)	1.55 (1.09)	0.046 (p=0.208)	0.113 (p=0.002)	0.079 (p=0.045)	0.126 (p=0.001)
HADS-A score, mean (SD)	4.49 (3.10)	−0.007 (p=0.842)	−0.114 (p=0.002)	−0.103 (p=0.011)	−0.001 (p=0.978)
HADS-D score, mean (SD)	2.51 (2.07)	−0.014 (p=0.704)	0.086 (p=0.021)	0.086 (p=0.032)	0.104 (p=0.004)
Smoking status		−0.018 (p=0.614)	0.477 (p<0.001)	0.098 (p=0.014)	0.191 (p<0.001)
Never smoked, number (%)	372 (49.1)				
Ex-smoker, number (%)	326 (43.0)				
Current smoker, number (%)	60 (7.92)				
Number of days alcohol is consumed per week, mean (SD)	2.70 (2.68)	0.015 (p=0.672)	0.133 (p=0.004)	0.130 (p=0.001)	−0.031 (p=0.397)
Time taken to walk 6 metres, mean (SD)	4.27 (1.10)	−0.025 (p=0.486)	0.090 (p=0.016)	0.033 (p=0.407)	0.128 (p=0.004)
Attitudes to ageing					
Physical change, mean (SD)	28.0 (5.11)	−0.023 (p=0.525)	−0.178 (p<0.001)	−0.123 (p=0.002)	−0.206 (p<0.001)
Psychosocial loss, mean (SD)	15.2 (4.79)	0.003 (p=0.938)	0.085 (p=0.023)	0.012 (p=0.628)	0.126 (p=0.005)
Psychological growth, mean (SD)	28.3 (4.33)	−0.005 (p=0.890)	−0.033 (p=0.386)	−0.023 (p=0.567)	−0.067 (p=0.065)
Brain-PAD, mean (SD)	1.38 (8.43)	0.038 (p=0.349)	0.198 (p<0.001)	–	0.142 (p=0.001)
Telomere length, mean (SD)	3971.41 (733.0)	–	0.046 (p=0.227)	0.038 (p=0.349)	0.0001 (p=0.998)
Accelerated DNA methylation GrimAge, mean (SD)	−0.28 (4.75)	0.046 (p=0.227)	–	0.198 (p<0.001)	0.209 (p<0.001)
Died during follow-up, number (%)	147 (19.4)	0.0001 (p=0.998)	0.209 (p<0.001)	0.142 (p=0.001)	–

*Correlations with social class are based on the six-category occupational social class variable.

Brain-PAD, brain-predicted age difference; DNA, deoxyribonucleic acid; HADS-A, Hospital Anxiety and Depression Scale, anxiety score; HADS-D, Hospital Anxiety and Depression Scale, depression score.

## Attitudes to ageing and ageing biomarkers

Table 2 shows the regression coefficients for each ageing biomarker according to an SD higher score for each domain of the AAQ. Coefficients are shown adjusted first for age and sex, then with additional separate adjustment for education and social class, smoking and frequency of alcohol consumption, chronic disease and walking speed, anxiety and depression symptoms and finally all covariates together. Looking first at log leucocyte telomere length, there were no significant associations between any of the attitude to ageing domain scores and this biomarker.

**Table 2:** Regression coefficients (95% CI) of ageing biomarkers according to standardised scores on attitudes to ageing scales at age 72 years

Attitudes to ageing scales, per SD	Leucocyte telomere length* (n=758)	Accelerated DNA methylation GrimAge† (n=715)	Brain-predicted age difference (n=622)
Physical change			
Adjusted for age and sex	−0.003 (−0.009 to 0.003) p=0.272; p^FDR^=0.637	−0.725 (−1.03 to –0.418) p<0.001; p^FDR^=0.001	−0.871 (−1.513 to –0.229) p=0.008; p^FDR^=0.062
Adjusted for age, sex, education and social class	−0.003 (−0.009 to 0.003) p=0.287; p^FDR^=0.637	−0.689 (−1.00 to –0.380) p<0.001; p^FDR^=0.001	−0.894 (−1.538 to –0.225) p=0.007; p^FDR^=0.062
Adjusted for age, sex, smoking and alcohol	−0.004 (−0.009 to 0.002) p=0.220; p^FDR^=0.625	−0.467 (−0.721 to –0.211) p<0.001; p^FDR^=0.001	−0.804 (−1.45 to –0.159) p=0.015; p^FDR^=0.101
Adjusted for age, sex, chronic disease and walking speed	−0.003 (−0.009 to 0.003) p=0.387; p^FDR^=0.756	−0.520 (−0.842 to –0.197) p=0.002; p^FDR^=0.021	−0.657 (−1.323 to –0.010) p=0.054; p^FDR^=0.224
Adjusted for age, sex, anxiety and depression symptoms	−0.004 (−0.001 to 0.003) p=0.255; p^FDR^=0.637	−0.703 (−1.03 to –0.373) p<0.001; p^FDR^=0.001	−0.672 (−1.346 to 0.001) p=0.050; p^FDR^=0.224
Multivariable-adjusted‡	−0.004 (−0.010 to 0.003) p=0.287; p^FDR^=0.637	−0.289 (−0.568 to –0.010) p=0.042; p^FDR^=0.206	−0.456 (−1.15 to 0.240) p=0.199; p^FDR^=0.625
Psychosocial loss			
Adjusted for age and sex	0.001 (−0.005 to 0.006) p=0.838; p^FDR^=0.935	0.370 (0.055 to 0.684) p=0.021; p^FDR^=0.126	0.127 (−0.514 to 0.768) p=0.697; p^FDR^=0.867
Adjusted for age, sex, education and social class	0.001 (−0.005 to 0.006) p=0.862; p^FDR^=0.935	0.335 (0.021 to 0.648) p=0.036; p^FDR^=0.194	0.156 (−0.488 to 0.801) p=0.634; p^FDR^=0.867
Adjusted for age, sex, smoking and alcohol	0.001 (−0.005 to 0.006) p=0.775; p^FDR^=0.930	0.098 (−0.162 to 0.358) p=0.460; p^FDR^=0.803	0.059 (−0.588 to 0.705) p=0.858; p^FDR^=0.934
Adjusted for age, sex, chronic disease and walking speed	0.001 (−0.006 to 0.006) p=0.937; p^FDR^=0.955	0.215 (−0.101 to 0.531) p=0.181; p^FDR^=0.611	−0.055 (−0.703 to 0.593) p=0.869; p^FDR^=0.935
Adjusted for age, sex, anxiety and depression symptoms	0.001 (−0,007 to 0.007) p=0.883; p^FDR^=0.935	0.325 (−0.039 to 0.689) p=0.080; p^FDR^=0.309	−0.260 (−0.984 to 0.465) p=0.482; p^FDR^=0.803
Multivariable-adjusted‡	0.001 (−0.006 to 0.007) p=0.876; p^FDR^=0.935	−0.010 (−0.308 to 0.282) p=0.931; p^FDR^=0.955	−0.389 (−1.119 to 0.340 p=0.295; p^FDR^=0.637
*Psychological growth*			
Adjusted for age and sex	−0.001 (−0.007 to 0.004) p=0.642; p^FDR^=0.868	−0.072 (−0.385 to 0.240) p=0.707; p^FDR^=0.867	−0.439 (−1.13 to 0.250) p=0.212; p^FDR^=0.625
Adjusted for age, sex, education and social class	−0.002 (−0.007 to 0.004) p=0.602; p^FDR^=0.868	−0.129 (−0.441 to 0.182) p=0.417; p^FDR^=0.776	−0.421 (−1.11 to 0.272) p=0.234; p^FDR^=0.632
Adjusted for age, sex, smoking and alcohol	−0.002 (−0.008 to 0.004) p=0.491; p^FDR^=0.803	0.113 (−0.146 to 0.371) p=0.392; p^FDR^=0.756	−0.352 (−1.05 to 0.343) p=0.320; p^FDR^=0.665
Adjusted for age, sex, chronic disease and walking speed	−0.001 (−0.007 to 0.004) p=0.678; p^FDR^=0.868	−0.069 (−0.376 to 0.239) p=0.662; p^FDR^=0.868	−0.473 (−1.16 to 0.213) p=0.176; p^FDR^=0.610
Adjusted for age, sex, anxiety and depression symptoms	−0.001 (−0.007 to 0.004) p=0.646; p^FDR^=0.868	0.003 (−0.317 to 0.323) p=0.986; p^FDR^=0.986	−0.219 (-0.925, 0.487) p=0.543; p^FDR^=0.837
Multivariable-adjusted‡	−0.002 (−0.008 to 0.004) p=0.490; p^FDR^=0.803	−0.075 (−0.188 to 0.338) p=0.575; p^FDR^=0.827	−0.191 (-0.906 to 0.525) p=0.601; p^FDR^=0.868

*All analyses of leucocyte telomere length were also adjusted for white blood cell counts.

†All analyses of accelerated DNA methylation GrimAge were adjusted for white blood cell counts.

‡Adjusted for age, sex, educational attainment, social class, total number of chronic diseases, smoking status, frequency of alcohol consumption, time taken to walk 6 metres, HADS anxiety score, HADS depression score.

§p^FDR^ is the p value corrected for multiple comparisons using the False Discovery Rate.

HADS, Hospital Anxiety and Depression Scale.

Turning next to AgeAccelGrim, more positive attitudes to physical change were associated with being biologically younger as measured by this biomarker in the age-adjusted and sex-adjusted model: for an SD higher score in attitudes to physical change, AgeAccelGrim was lower by 0.725 (95% CI 1.03 to 0.418) of a year. Adjustment for education and social class or anxiety and depression symptoms had only minor effects on this association, but it was attenuated by 36% after adjustment for smoking and frequency of alcohol consumption, and by 28% after adjustment for chronic disease and walking speed, although in each case the association remained significant after correction for multiple testing. In the final model adjusting for all covariates, the relationship was attenuated by 60%: for an SD higher score in attitudes to physical change, AgeAccelGrim was lower by 0.289 (95% CI 0.568 to 0.010) of a year. This was not significant after correction for multiple testing. Having a more positive attitude to psychosocial loss, as indicated by a lower score on this domain, was associated with a slightly lower AgeAccelGrim in the age-adjusted and sex-adjusted model, but this was not significant after correction for multiple testing. There was no association between attitudes to psychological growth and AgeAccelGrim.

Looking finally at brain-PAD, in the age-adjusted and sex-adjusted model, an SD higher score for attitudes to physical change was associated with a brain-PAD that was lower by 0.871 (95% CI 1.513 to 0.229) of a year. This ceased to be significant after correction for multiple testing. This association was slightly strengthened by adjustment for education and social class but remained non-significant after correction for multiple testing. Adjustment for either chronic disease and walking speed or anxiety and depression symptoms had the strongest attenuating effects, reducing the association by 25% or 22%, respectively. In the final model adjusting for all covariates, the relationship was attenuated by 48% and no longer significant: for an SD higher score in attitudes to physical change, brain-PAD was lower by 0.456 (95% CI −1.15 to 0.240) of a year. Attitudes to psychosocial loss and psychological growth were not significantly associated with brain-PAD.

## Attitudes to ageing and mortality

Tables 3 and 4 show the HRs (95% CI) for death during the follow-up period according to attitudes to ageing. Estimates are shown adjusted first for age and sex, then for AgeAccelGrim (table 3) or brain-PAD (table 4), then for education and social class, smoking and frequency of alcohol consumption, chronic disease and walking speed and anxiety and depression symptoms, separately and finally all together. In order to see the extent to which any effect of either AgeAccelGrim or brain-PAD on the relationships between attitudes to ageing and mortality might be explained by the covariates, we also show the HRs adjusted for each type of covariate plus either AgeAccelGrim or brain-PAD. We have not included a similar table showing estimates adjusted for leucocyte telomere length as this was not associated with mortality or attitudes to ageing in this sample (tables 1 and 2).

**Table 3:** HRs (95% CI) of death from all causes according to standardised scores on attitudes to ageing scales at age 72 years, adjusted for accelerated DNA methylation GrimAge (AgeAccelGrim) and covariates (N=715)

Adjustments	Physical change	†p/p^FDR^	Psychosocial loss	†p/p^FDR^	Psychological growth	†p/p^FDR^
Age and sex	0.66 (0.56 to 0.78)	<0.001/0.0003	1.28 (1.09 to 1.50)	0.002/0.004	0.87 (0.74 to 1.02)	0.089/0.120
Age, sex and AgeAccelGrim	0.71 (0.61 to 0.84)	<0.001/0.0003	1.23 (1.05 to 1.44)	0.0011/0.020	0.89 (0.75 to 1.03)	0.110/0.140
Age, sex, education and social class	0.67 (0.57 to 0.79)	<0.001/0.0003	1.25 (1.07 to 1.47)	0.005/0.009	0.84 (0.72 to 0.99)	0.040/0.064
Age, sex, education and social class plus AgeAccelGrim	0.72 (0.62 to 0.85)	<0.001/0.0003	1.21 (1.03 to 1.42)	0.020/0.033	0.86 (0.73 to 1.01)	0.065/0.097
Age, sex, smoking and alcohol	0.69 (0.58 to 0.81)	<0.001/0.0003	1.23 (1.05 to 1.44)	0.011/0.020	0.87 (0.74 to 1.02)	0.088/0.119
Age, sex, smoking and alcohol plus AgeAccelGrim	0.71 (0.60 to 0.83)	<0.001/0.0003	1.22 (1.04 to 1.42)	0.016/0.029	0.86 (0.75 to 1.01)	0.068/0.100
Age, sex, chronic disease and walking speed	0.73 (0.61 to 0.86)	<0.001/.0003	1.18 (1.01 to 1.39)	0.043/0.067	0.87 (0.75 to 1.02)	0.092/0.121
Age, sex, chronic disease plus AgeAccelGrim	0.76 (0.64 to 0.91)	0.001/0.002	1.16 (0.99 to 1.37)	0.075/0.106	0.88 (0.76 to 1.04)	0.138/0.168
Age, sex, anxiety and depression	0.67 (0.56 0.80)	<0.001/0.003	1.25 (1.03 to 1.51)	0.021/0.034	0.90 (0.77 to 1.07)	0.233/0.244
Age, sex, anxiety and depression plus AgeAccelGrim	0.72 (0.60 to 0.86)	<0.001/0.003	1.21 (1.00 to 1.45)	0.048/0.074	0.90 (0.77 to 1.06)	0.225/0.240
All*	0.76 (0.64 to 0.91)	0.002/0.004	1.15 (0.96 to 1.39)	0.128/0.159	0.87 (0.74 to 1.03)	0.104/0.135

*Age, sex, AgeAccelGrim, educational attainment, social class, total number of chronic diseases, time taken to walk 6 metres, smoking status, frequency of alcohol consumption, HADS anxiety score, HADS depression score.

†p^FDR^ is the p value corrected for multiple comparisons using the False Discovery Rate.

HADS, Hospital Anxiety and Depression Scale.

**Table 4:** HRs (95% CI) of risk of death from all causes according to standardised scores for attitudes to ageing at age 72 years, adjusted for brain-PAD and covariates (n=622)

Adjustments	Attitudes to ageing scales (per SD)					
	Physical change	†p/p^FDR^	Psychosocial loss	†p/p^FDR^	Psychological growth	†p/p^FDR^
Age and sex	0.64 (0.53 to 0.77)	<0.001/0.003	1.44 (1.21 to 1.70)	<0.001/0.003	0.88 (0.73 to 1.06)	0.169/0.195
Age, sex and brain-PAD	0.65 (0.54 to 0.78)	<0.001/0.003	1.42 (1.20 to 1.69)	<0.001/0.003	0.89 (0.74 to 1.07)	0.217/0.235
Age, sex and education	0.65 (0.54 to 0.79)	<0.001/0.003	1.41 (1.18 to 1.67)	<0.001/0.003	0.84 (0.70 to 1.02)	0.076/0.106
As above plus brain-PAD	0.67 (0.56 to 0.81)	<0.001/0.003	1.39 (1.17 to 1.66)	<0.001/0.003	0.84 (0.70 to 1.02)	0.085/0.116
Age, sex, smoking and alcohol	0.66 (0.55 to 0.80)	<0.001/0.003	1.36 (1.15 to 1.61)	<0.001/0.003	0.88 (0.73 to 1.06)	0.165/0.194
Age, sex, smoking and alcohol plus brain-PAD	0.68 (0.56 to 0.81)	<0.001/0.003	1.35 (1.14 to 1.60)	0.001/0.002	0.89 (0.74 to 1.06)	0.194/0.133
Age, sex, chronic disease and walking speed	0.69 (0.57 to 0.84)	<0.001/0.003	1.34 (1.12 to 1.60)	0.001/0.002	0.87 (0.73 to 1.05)	0.140/0.168
Age, sex, chronic disease and walking speed plus brain-PAD	0.71 (0.58 to 0.86)	<0.001/0.003	1.34 (1.12 to 1.60)	0.001/0.002	0.88 (0.73 to 1.06)	0.180/0.204
Age, sex, anxiety and depression	0.68 (0.56 to 0.82)	<0.001/0.003	1.36 (1.11 to 1.67)	0.003/0.006	0.93 (0.77 to 1.13)	0.477/0.492
Age, sex, anxiety and depression plus brain-PAD	0.68 (0.56 to 0.82)	<0.001/0.003	1.37 (1.12 to 1.67)	0.002/0.004	0.94 (0.78 to 1.13)	0.489/0.497
All*	0.76 (0.62 to 0.92)	0.006/0.011	1.27 (1.04 to 1.55)	0.020/0.033	0.88 (0.73 to 1.07)	0.196/0.216

*Age, sex, brain-PAD, educational attainment, total number of chronic diseases, time taken to walk 6 metres, smoking status, frequency of alcohol consumption, HADS anxiety score, HADS depression score.

†p^FDR^ is the p value corrected for multiple comparisons using the False Discovery Rate.

Brain-PAD, brain-predicted age difference; HADS, Hospital Anxiety and Depression Scale.

In participants with data on AgeAccelGrim (table 3), participants with a more positive attitude to physical change had a markedly lower risk of mortality after adjusting for age and sex: for an SD higher score, the HR (95% CI) was 0.66 (0.56 to 0.78); this remained significant after correction for multiple testing. Adjustment for AgeAccelGrim attenuated this by 14.7%. Adjustment for each type of covariate in turn had minor attenuating effects on the age-adjusted and sex-adjusted effect size. When AgeAccelGrim was added to each of these models it had small attenuating effects, suggesting its effect is not explained entirely by these covariates. After full adjustment for AgeAccelGrim and all other covariates, having a more positive attitude to physical change remained associated with lower mortality after correction for multiple testing: for an SD higher score, the HR (95% CI) was 0.76 (0.64 to 0.91). Having a more positive attitude to psychosocial loss (indicated by lower scores) was also associated with lower mortality in age-adjusted and sex-adjusted models: for an SD higher score, the HR was 1.28 (1.09 to 1.50); this was significant after correction of multiple testing. This was attenuated by 17.9% by adjustment for AgeAccelGrim. Adjustment for each type of covariate in turn had some attenuating effects on the age-adjusted and sex-adjusted effect size, with chronic disease and walking speed having the strongest effect, weakening the association by 35.7%. After full adjustment for AgeAccelGrim and all covariates, the association was attenuated by 46.4% and was not significant. People with a more positive attitude to psychological growth also had a lower risk of death in age-adjusted and sex-adjusted models: for an SD higher score, the HR was 0.87 (0.74 to 1.02), but this was not significant. Adjustment for AgeAccelGrim attenuated this by 15.4%.

In people with data on brain-PAD, the associations between attitudes to ageing and mortality were similar to those described above. In this sample, having a more positive attitude to physical change and to psychosocial loss were both associated with lower mortality and these associations remained significant after adjustment for all covariates after correction for multiple testing. However, the amount explained by brain-PAD was tiny: adjustment for this attenuated the age-adjusted and sex-adjusted HRs between attitudes to physical change or psychosocial loss and mortality by 2.8% and 4.5%, respectively.

## Discussion

In these older adults, having a more positive attitude to physical change was associated with being biologically younger as measured by AgeAccelGrim and brain-PAD in age-adjusted and sex-adjusted models. These associations were attenuated by adjustment for covariates, and neither was significant after adjustment for all covariates and correction for multiple testing. Attitudes to psychosocial loss or psychological growth were not significantly associated with AgeAccelGrim or brain-PAD. None of the three attitudes to ageing domain scores were associated with leucocyte telomere length. People with more positive attitude to physical change had a lower risk of death during follow-up; being biologically younger as measured by AgeAccelGrim and brain-PAD accounted for at most 14.7% or 2.8%, respectively of these relationships. Having a more positive attitude to psychosocial loss was also associated with a lower risk of death, but only in the subsample with data on brain-PAD.

There is little prior evidence about the relationships between attitudes to ageing and ageing biomarkers. Contrary to the findings here, one study found that positive attitudes to ageing were associated with having a longer telomere length 4 years later.^15^ This study did not use a continuous measure of telomere length but created a binary indicator of whether telomere length was ‘normal length’ or ‘shorter length’ and did not examine different attitude domains separately. To our knowledge, this is the first study to examine the associations between attitudes to ageing and brain-PAD and AgeAccelGrim. A recent study found that younger subjective age is associated with less-advanced brain ageing.^16^ This is consistent with our findings using a broader measure of attitudes of ageing.

Other studies which found associations between attitudes to ageing and mortality^10 11^ used the ‘Attitudes Toward Ageing’ subscale from Philadelphia Geriatric Center Morale Scale (PGCMS).^33^ These findings are consistent with the present study since there is considerable overlap between items in this scale and the physical change domain of the AAQ.^1^ Items from the PGCMS and the physical change domain of the AAQ tend to focus on personal experience of ageing (eg, “My health is better than I expected for my age”^1^), while items from the psychosocial loss and psychological growth AAQ domains focus on attitudes towards the ageing process more generally (eg, ‘Old age is a time of loneliness’^1^). To the best of our knowledge, this the first study to examine the role of biomarkers of ageing in explaining the relationship between attitudes to ageing and mortality.

This study has several strengths. The AAQ has been validated on samples of older people from several countries.^34 35^ A variety of potential confounding or mediating factors were controlled for. Statistics on variability of the biomarkers of ageing, either at wave 2 or over successive waves of data collection, show they are reliable measures. One major limitation is that the relationship between attitudes to ageing and ageing biomarkers was analysed cross-sectionally, making it impossible to determine the direction of relationships. It is important to note that although we included smoking as a covariate, DNA methylation-based surrogates for smoking are one of the components of DNA methylation GrimAge.^27^ Indeed, as smoking status is highly correlated with AgeAccelGrim (rho=0.484, p<0.001), adjustment for smoking complicates the interpretation of the relationship between AgeAccelGrim and attitudes to ageing. Although we took account of the number of diagnoses of chronic illnesses participants reported, no data were collected specifically on diagnoses of chronic obstructive pulmonary disease and we had no information on hospitalisations in the past year which might have provided an indicator of disease severity. The LBC1936 cannot be considered representative of the general population of Scotland born in that year as they have continued to live in the Lothian area. They also had a higher mean childhood intelligence than the overall population, and are likely to have higher educational attainment and more advantaged social class.

The association between attitudes towards physical change and mortality could reflect the fact that people who have poor health are both more likely to die and more likely to report physical decline on the AAQ. More advanced methylation ageing is manifest as impaired physical function^36^ and it can be assumed that negative attitudes towards physical change simply represent an awareness of this impaired function. However, the association remained significant after controlling for chronic disease and walking speed. Levy argues that people’s attitudes to ageing are not a proxy for physical health but predict mortality by becoming a self-fulfilling prophecy.^2^ Indeed, previous research suggests that attitudes to ageing measured many years earlier predict health and longevity in older age.^4 9 10 37^

This study found limited evidence that attitudes to ageing are linked with ageing biomarkers. More positive attitudes towards physical change were associated with being biologically younger, as indicated by AgeAccelGrim and brain-PAD in age-adjusted and sex-adjusted models, but these associations were not significant after full adjustment for confounding or mediating factors and correction for multiple testing. Positive attitudes to physical change were associated with a reduced mortality risk after adjustment for confounding or mediating factors, but AgeAccelGrim or brain-PAD explained very little of this association. Since trials have shown that improving people’s attitudes to ageing benefits physical health and function,^38–40^ such initiatives may also decelerate biological ageing and reduce mortality. Further research should explore the longitudinal effects of attitudes to ageing on ageing biomarkers and examine whether other factors (eg, cardiovascular stress) explain the relationship between attitudes to physical change and mortality.

**What is already known on this subject**

- Older people with more negative attitudes to ageing have an increased risk of adverse health outcomes, including earlier death.
- The underlying mechanisms are poorly understood. One possibility may be that people with more negative attitudes to ageing are biologically older.

**What this study adds**

- Older people with a more negative attitude to physical change were biologically older as defined by accelerated DNA methylation GrimAge and brain-predicted age difference in age-adjusted and sex-adjusted models.
- These associations were no longer significant after further adjustment for potential confounding or mediating variables and correction for multiple testing.
- Having a more positive attitude to physical change was associated with reduced risk of death, but biomarkers of ageing, and other covariates, explained little of this association.
