# Supplementary material for: Attitudes to ageing, biomarkers of ageing and mortality: the Lothian Birth Cohort 1936
Source: J Epidemiol Community Health. 2020 Jan 28;74(4):377–83. doi: 10.1136/jech-2019-213462 (PMC7079194; doi:10.1136/jech-2019-213462)
Supplement: Supplementary data [file jech-2019-213462supp001.pdf]

Variability of the biomarkers of ageing

Measures of brain-predicted age are only currently available from one wave of data collection (wave 2). These measures show very high test-retest reliability; intraclass correlation coefficient = 0.97 [95% CI: 0.92, 0.99] for same scanner, 0.92 [0.74, 0.98] for two different scanners.[1]

Leukocyte telomere length measurements were made in quadruplicate and the mean of the measurements used. At wave 2 the intra-assay coefficient of variation was 2.7% and the inter-assay coefficient of variation was 5.1%. The correlations between telomere length measured at wave 2 with telomere length measured at the subsequent three waves of data collection (each 3 years apart) range from 0.83 (wave 3) to 0.64 (wave 5). The intraclass coefficients for telomere measurements made over five waves of data collection was 0.52 (single measures) and 0.84 (average measures).

Accelerated DNA methylation GrimAge shows considerable reliability over time: the intraclass coefficient for measurements made over four waves of data collection was 0.83. No information is available on period-free reliability of DNA methylation GrimAge, but its high reliability over time gives a lower-bound estimate of its period-free reliability.

Reference

1. Cole JH, Poudel RPK, Tsagkrasoulis D, et al. Predicting brain age with deep learning from raw imaging data results in a reliable and heritable biomarker. *NeuroImage.* 2017;**163**:115-24.
